# Supplementary material for: Garrano Horses Perceive Letters of the Alphabet on a Touchscreen System: A Pilot Study
Source: Animals (Basel). 2022 Dec 12;12(24):3514. doi: 10.3390/ani12243514 (PMC9774258; doi:10.3390/ani12243514)
Supplement: Supplementary file 1 [file animals-12-03514-s001.zip › animals-2053942-Supplementary.pdf]

animals

MDPI

Supplementary Material S3：Document explaining the details of the study. **A-1 Information on individual horses**

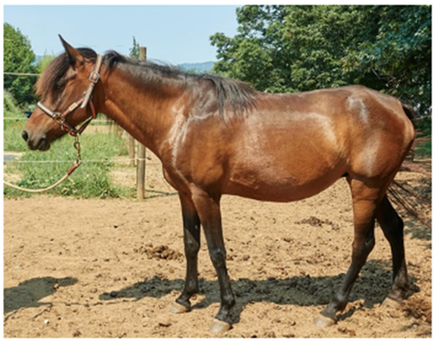 Full body portrait of Flore	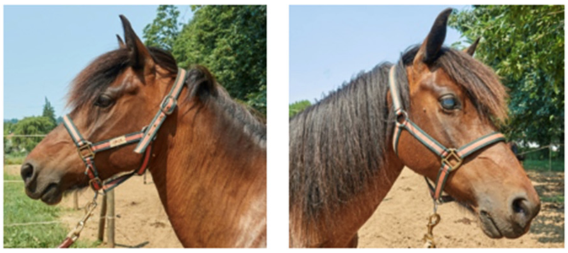 Head portrait of Flore (left eye intact, right eye blind)
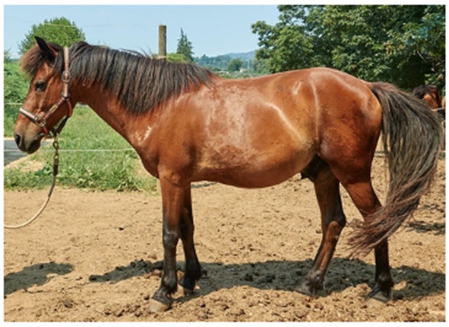 Full body portrait of Boneko	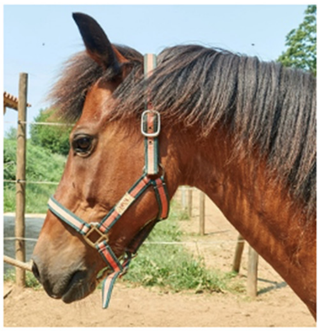 Head portrait of Boneko
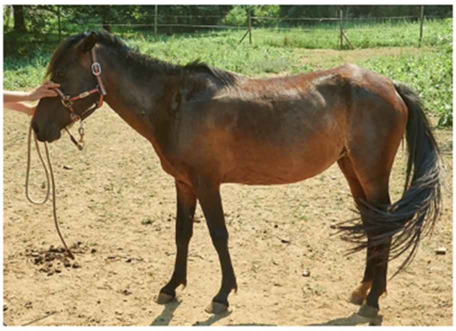 Full body portrait of Noven	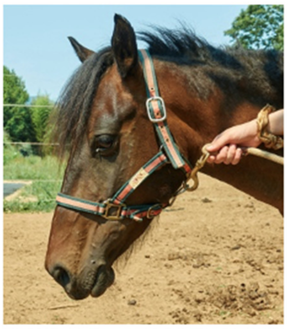 Head portrait of Noven
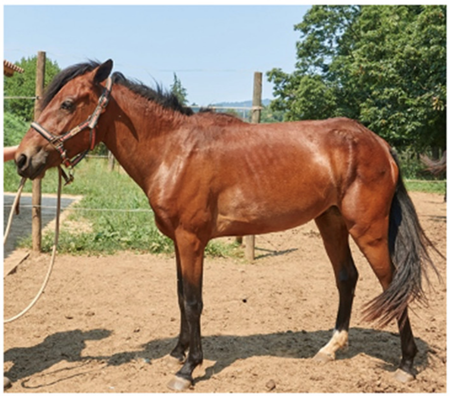 Full body portrait of Petala	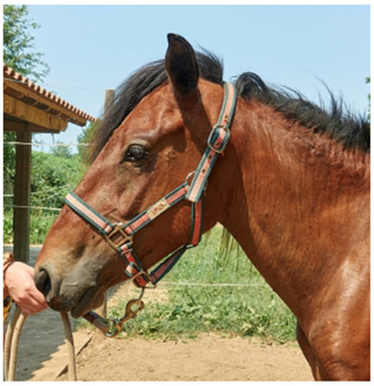 Head portrait of Petala

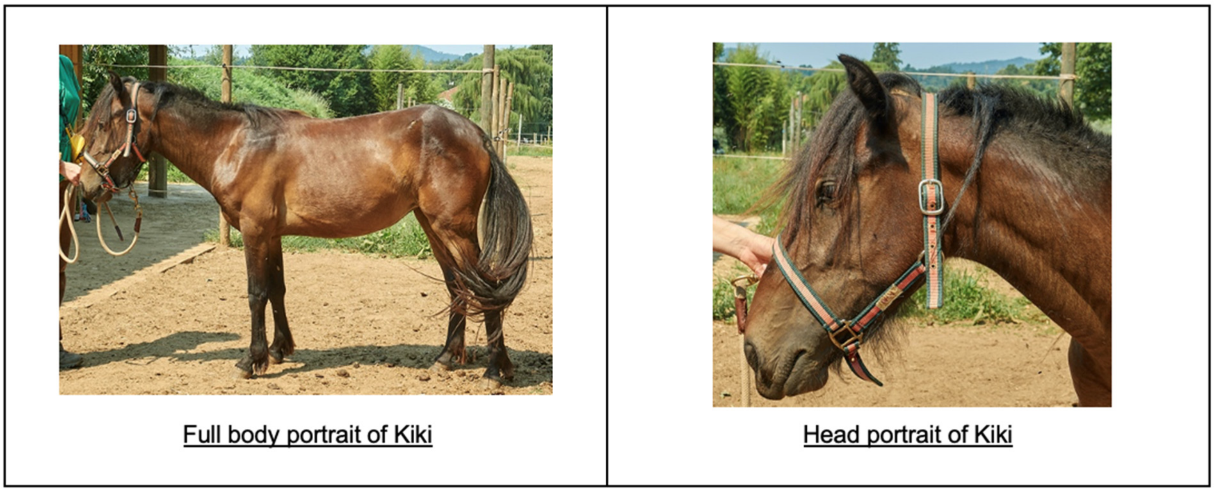

Figure S1. Individual photos of the five horse participants. Full body portraits and Head portraits (Photos by Clara Lynn Schubert and Barbara Ryckewaert).

A-2 The apparatus behind the screen

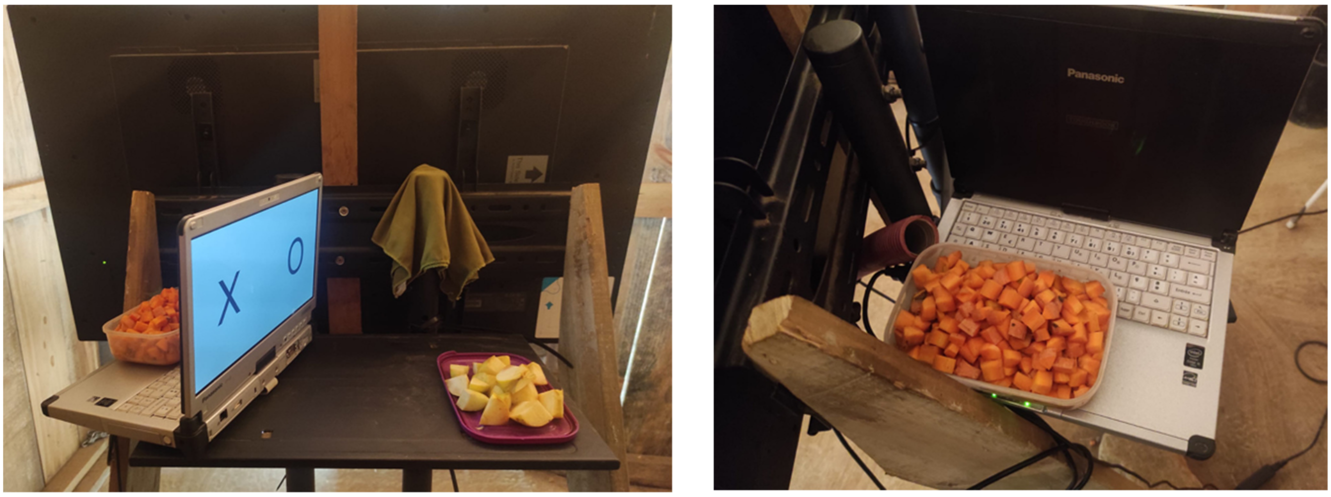

Figure S2. Behind the screen. (Left): The experiment setup behind the touchscreen. (Right): The experimental setup behind the touchscreen (from the perspective of the experimenter). (photos by Clara-Lynn Schubert)

A-3 Study environment: perceptual and cognitive testing of horses in a family setting

The five participant horses belong to the association named "O caminho do Gararno (The way of Garano)". The association is allied with The Camara Municipal de Viana do Castelo and shares responsibility for the daily care of horses. The research facility is called Garrano Horse Center (GHC). In GHC, horses live permanently outdoors and they are neither separated nor confined in the stables. Thus, horses are semi-free in their native climate near Serra d'Arga. The group of 5 horses simulates the family group (one-male unit, OMU) of feral horses. Through the intensive preliminary training over several months, each horse adapted to temporary separation from the group to go to the stall. Based on their free will, they participated in the touchscreen task. Top: enclosure, Center: group feeding on hay, Bottom: going to the touchscreen in the stall (Photos by Clara Lynn Schubert and Barbara Rychebaert).

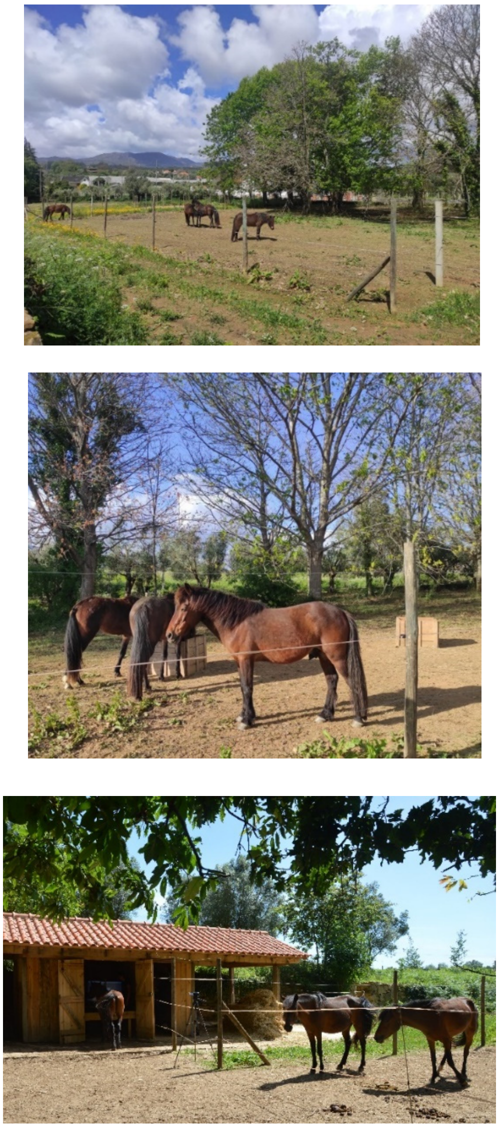

Figure S3. Study environment: perceptual and cognitive testing of horses in a family setting

A-4 Learning curves during training.

The change in accuracy (% correct) during the training sessions for each horse. These data show individual differences in discrimination learning in the five horses. They also illuminate what was easy or difficult for each horse. Each figure shows the learning curve to reach the criterion of 100% accuracy in two consecutive sessions for each size of X in discrimination learning (X1: 5 cm, X2: 8cm, and X3:15 cm).

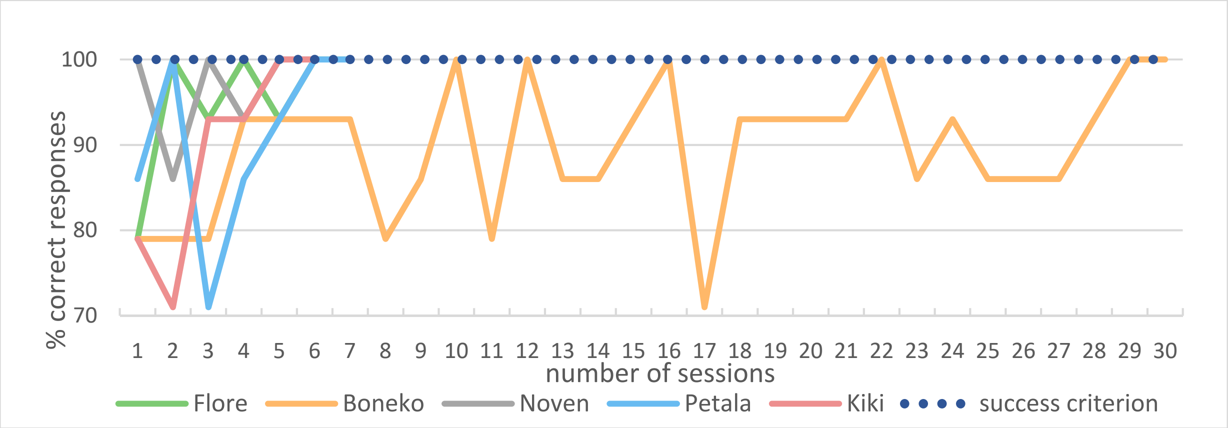

Figure S4-1. Accuracy (% correct responses) in ● vs X1 discrimination for each horse.

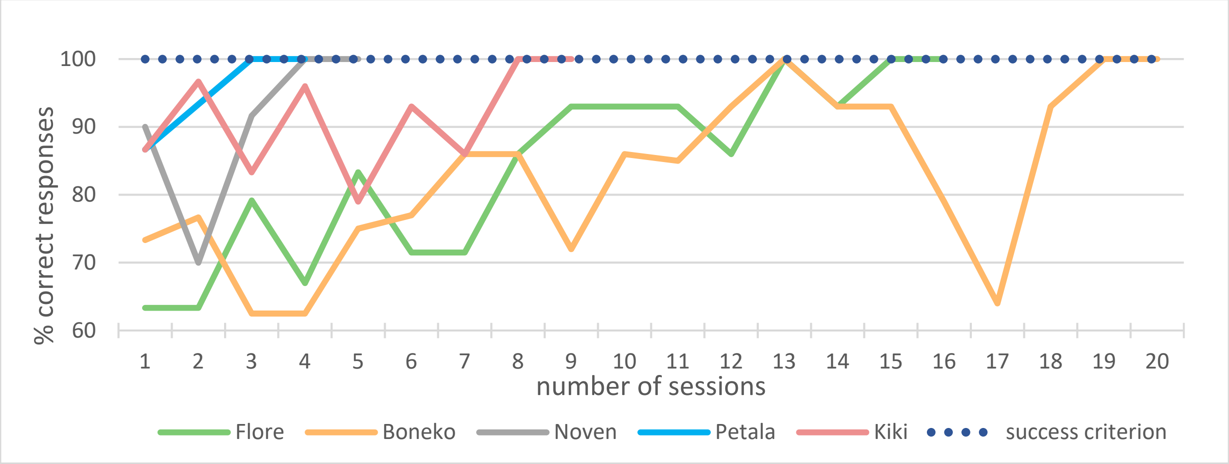

Figure S4-2. Accuracy (% correct responses) in ● vs X2 discrimination for each horse.

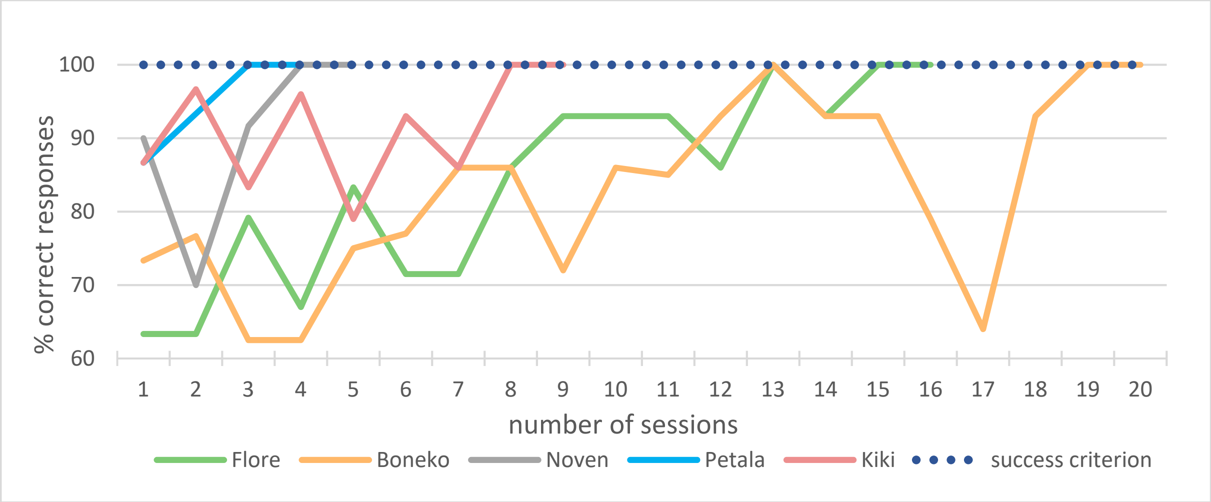

Figure S4-3. Accuracy (% correct responses) in ● vs X3 discrimination for each horse.

A-5 Individual MDS analysis

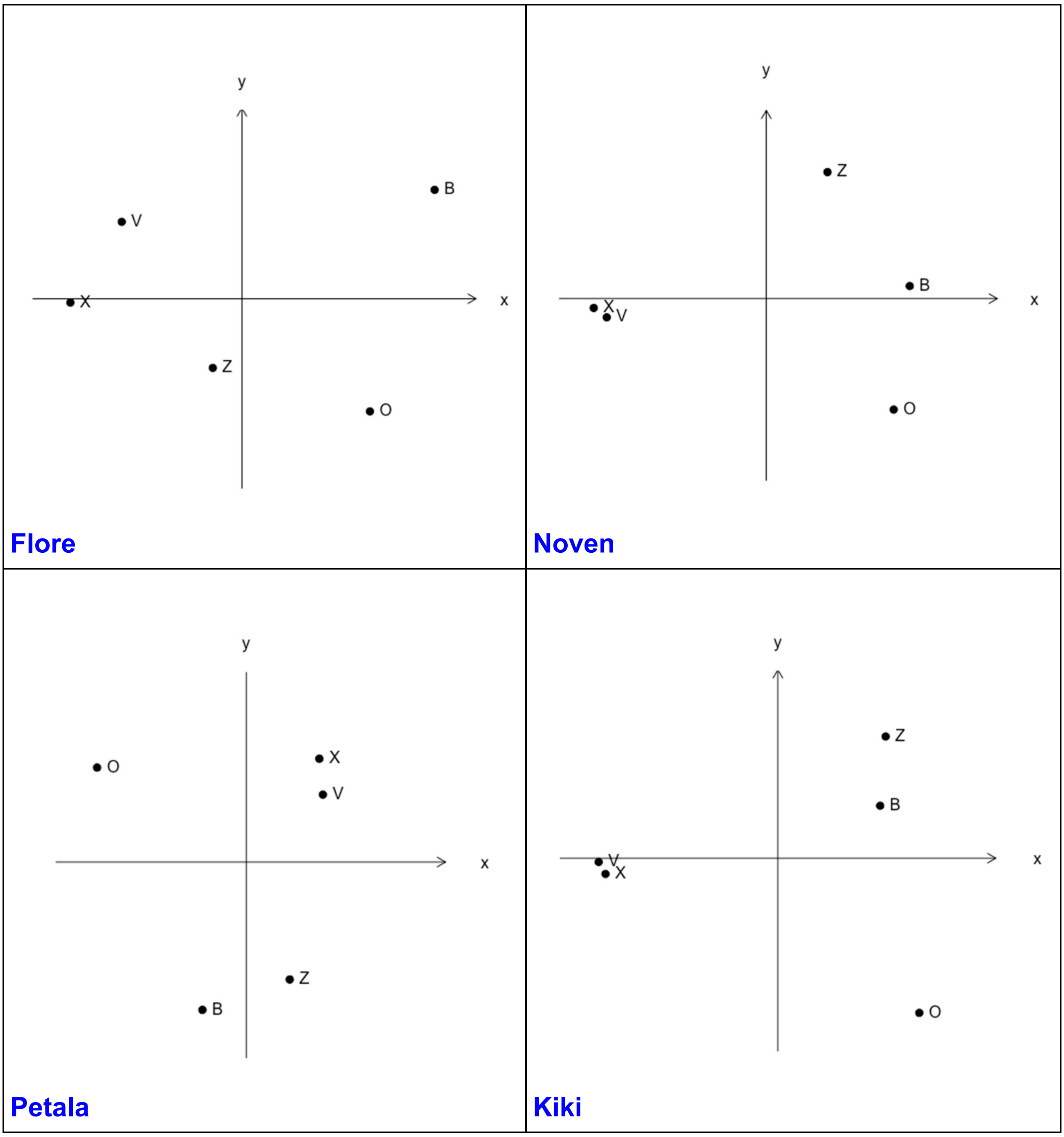

Figure S5. Perceived similarity among the five letters for each horse A two-dimensional solution of MDS was applied to the dissimilarity matrix for each horse.

A-6 Features

Shape features: Shapes can be clustered by their so-called "features" [56]. For example, this study focused on curved shapes: O and B, and shapes with diagonal lines: V, Z, and X. Table S6-1 shows the related features of these five letters. The present study adopted the six features (Vertical/Horizontal, Diagonal, Curvature, Closure, Open end, and Acute angle) used with dolphins, chimpanzees, and humans proposed by Tomonaga et al [46]. A 7th feature, "intersection," was added to differentiate shapes V and X [56].

Table S6-1 Features for shape discrimination. The present study used the following 7 features to discriminate the 5 letters of the alphabet. Some features are overlapped because of the small set of letters of the alphabet in the present study.

	Features						
Letter	Vertical/ Horizontal	Diagonal	Curvature	Closure	Open end	Acute angle	Intersection
O			✓	✓			
B	✓		✓	✓			✓
Z	✓	✓			✓	✓	
V		✓			✓	✓	
X		✓			✓	✓	✓

The relative contribution of shape features: To evaluate the relative contribution of each shape feature to the perceptual grouping, we calculated the mean percent of correct choices for pairs in which both stimuli shared the same individual features. These values were standardized using means and standard deviations (referred to as standardized dissimilarity) (see Figure A6-1). Note that the overall mean discrimination score was 80.4% (SD=13.7%), as shown in Table 2 in the main text. All of the features except "intersection" showed minus values, so the pairs that share those features were confusing for the horses. In particular, "Oblique/Diagonal lines", "Open end", and "Acute angle" were the most confusing. We cannot determine the contribution of each of these features separately in the present study because of the small number of letters. Thus, the letters X, V, and Z, with straight lines in common, were perceived as confusing by the horses. In terms of the letter pairs, six pairs (O vs. B, B vs. Z, Z vs. V, V vs. X, Z vs. X, except B vs. X) were confusing because they share some features (mean accuracy of 75.0%, SD=14.9%). In contrast, four other pairs (O vs. Z, O vs. V, O vs. X, B vs. V) were not confusing due to the absence of shared features (mean accuracy 88.5%, SD=4.7%). Although pair B vs. X shares the feature of "Intersection", this feature did not contribute to perceived similarity.

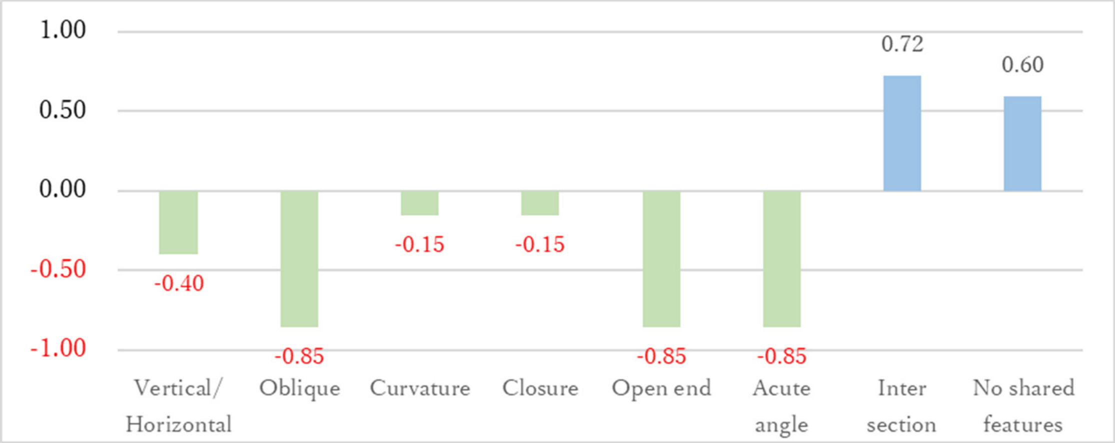

Figure S6-1. The relative contribution of each feature to perceptual similarities. The Y-axis shows the mean standardized dissimilarity. A smaller value indicates that letters sharing those features are perceived as more similar to one another. "No shared features" means letter pairs that do not share any of the 7 features: O vs. X, O vs. V, O vs. Z, and B vs. V.

A full list of shape features: As described, shapes can be clustered by a set of "features" [47, 48, 56]. There are 26 letters in the Roman alphabet. For further extension of the study on the perception of letters by humans and nonhuman animals, we tried to list the possible features to discriminate the letters of the alphabet (see Table A6-2). There are 26 letters so there are 26 x 25 / 2 = 325 possible pairs in total. Here, Schubert & Matsuzawa propose the 11 features that can discriminate 100 % of the possible pairs. The relative contribution of features can be expressed by the dissimilarity index based on the accuracy of discrimination as shown in Table 2 of this manuscript. Future studies of testing the confusion matrix of 26 letters will prove whether these features are really important or not. There might be a species difference in the relative contribution of features. For example, the terrestrial animal might be more sensitive to the feature of Vertical/Horizontal. In contrast, avian (birds) and arboreal (nonhuman primates) species might be insensitive to the feature because they are free to change their body orientation in the 3-dimensional space. The perceived similarity can be shown in the response latency as well. Suppose that the 2 letters are perceived as similar, the latency to make the same/different judgment should take a longer time.

Table S6-2. Letters of the Roman alphabet and possible "features" of visual perception. The present study used the 7 features to discriminate the 5 letters of the alphabet. Some features are overlapped because of the small set of 5 letters of the alphabet in the present study (highlighted by sky blue). To cover the full set of 26 letters, we proposed 11 features.

	Features										
Letter	Vertical/ Horizontal	Oblique/ Diagonal	Curvature	Closure	Open end	Acute angle	Right angle	Inter-section	Left/Right Symmetry	Top/Bottom symmetry	Wide breadth
A	✓	✓		✓	✓	✓					
B	✓		✓	✓				✓			
C			✓		✓					✓	
D	✓		✓								
E	✓				✓		✓			✓	
F	✓				✓		✓				
G	✓		✓		✓		✓				
H	✓				✓		✓		✓	✓	
I	✓				✓				✓	✓	
J	✓		✓		✓						
K	✓	✓			✓	✓		✓			
L	✓				✓		✓				
M	✓	✓			✓	✓			✓		✓
N	✓	✓			✓	✓					
O			✓	✓					✓	✓	
P	✓		✓	✓	✓						
Q		✓	✓	✓	✓			✓			
R	✓	✓	✓	✓	✓		✓	✓			
S			✓		✓						
T	✓				✓		✓		✓		
U	✓		✓		✓				✓		
V		✓			✓	✓			✓		
W		✓			✓	✓			✓		✓
X		✓			✓	✓		✓	✓	✓	
Y	✓	✓			✓	✓		✓	✓		
Z	✓	✓			✓	✓					

The 7 features by Tomonaga et al (2014) were highlighted in yellow while the additional feature proposed by the present study was highlighted in sky blue

There are 26 pairs of letters of the alphabet. Thus, there are 26 x 25 / 2 = 325 pairs among them.

Schubert & Matsuzawa 11 features listed above can discriminate all 325 out of 325 pairs (100 %).

Schubert & Matsuzawa 7 features used in the present study (Table 2) can discriminate 320 out of 325 pairs (98.5 %), except (E/F/H), (M/N), (V/W).

Tomonaga 7 features can discriminate 318 out of 325 pairs (97.8%), except (E/F/H), (M/N), (V/W/X)

A-7 Improvement of the apparatus

Other aspects could be improved in future studies besides increasing the number of participants. First is the touch detection sensitivity. The automated touch detection showed differential sensitivity to touches by the five horses. Manual validation of the horse touch response should be avoided and improved in the future. Second is the introduction of a universal feeder (BUF-310-P25, Bio-Medica) similar to Tomonaga et al (2015) [23]. This would reduce the experimenter's degree of involvement in the experiment. A bowl feeder can be improved too. Automatic detection of the carrot cube delivery can signal the end of the trial. The 5 seconds preset ITI should start not from the touch but from the food delivery to keep the real interval constant. The third is the reversal of S+ and S-: due to time-budget limitations, this was not tested in the present study. Fourth is the assessment of hearing. In the touch panel system, nose touch immediately resulted in auditory feedback (correct or error) (Methods 2-4-1). A previous study showed that older horses react less to sounds than younger ones [37]. Hearing might be critical for discrimination learning. A solution might be to add tactile feedback. Fifth is the sophistication of the assessment of vision. In addition to the menace reflex evaluation, other tests could provide more information on the participants' vision status and neurological states, such as the palpebral (blink) reflex, corneal reflex, and pupillary light reflex.
